# Effects of feeding high-moisture corn on growth performance and rumen metabolism in young Simmental bulls

**DOI:** 10.3389/fvets.2025.1688465

**Published:** 2026-03-11

**Authors:** Yu Gao, Kangyu Yao, Linhai Song, Wen Jiang, Min Yang, Yuxin Zhou, Subinur Abduali, Kadriya Abdurayimu, Wei Shao, Liang Yang, Wanping Ren

**Affiliations:** Xinjiang Key Laboratory of Meat and Milk Production Herbivore Nutrition, College of Animal Science, Xinjiang Agricultural University, Urumqi, China

**Keywords:** amino acid metabolism, economic benefits, growth performance, rumen microorganism, Simmental bull

## Abstract

**Introduction:**

This study evaluated the effects of different dietary inclusion levels of high-moisture corn (HMC) on the growth performance and rumen metabolism of young Simmental bulls.

**Methods:**

Thirty-two young Simmental bulls (243.99 ± 13.52 kg) were randomly allocated to one of four dietary treatments: (1) control group (HMC0), fed a basal diet; (2) HMC15 group, where 15% of the conventional corn in the diet was replaced with HMC; (3) HMC30 group, with a 30% replacement level of HMC; and (4) HMC45 group, with a 45% replacement level of HMC. Live body weights were recorded from all bulls on days 0, 30, 60, and 120. On day 120, rumen fluid samples were also obtained, filtered, and properly preserved at −80 °C for later determination of rumen fermentation and nutrient metabolism profiles.

**Results:**

The results demonstrated that on day 120, the body weights of the HMC30 and HMC45 groups were significantly higher than that of the HMC0 group (*p* < 0.05). Dietary HMC supplementation significantly improved the average daily gain (ADG) and profit of young Simmental bulls (*p* < 0.05). In contrast, the HMC45 group showed significantly lower acetic acid and total volatile fatty acids (total VFA) concentrations compared to the HMC0 group, while the lactic acid (LA) concentration was the opposite(*p* < 0.05). Furthermore, a trend of decreasing ruminal pH was observed with increasing HMC inclusion levels. Concurrently, HMC feeding significantly enhanced the alpha diversity of the rumen microbiota. Rumen metabolomics revealed distinct metabolic alterations in the HMC45 group, identifying 52 differential metabolites—including 1,2-Dioleoyl-sn-Glycero-3-Phosphocholine (DOPC), PC, taurine, and L-glutamic acid—which were primarily involved in key pathways such as glycerophospholipid metabolism, bile acid metabolism, and glutamate metabolism.

**Discussion:**

In conclusion, the 45% HMC substitution level significantly enhanced the production performance of young Simmental bulls by modulating key metabolites involved in glycerophospholipid metabolism, bile secretion, and amino acid metabolism, thereby enriching rumen microbial diversity and ultimately improving growth outcomes

## 1 Introduction

HMC is an energy feed with silage characteristics, produced by crushing and compacting corn kernels with 25%–35% moisture content, incorporating microbial agents, and storing in silage pits or bale-wrapped stacks for long-term preservation of nutritional components (1, 2). As the primary energy source in livestock feed, corn contains a starch-protein matrix that reduces ruminants’ starch digestibility and leads to wastage. HMC, however, breaks down this matrix, thereby improving digestion efficiency and minimizing feed loss (3–6). Additionally, HMC demonstrates advantages such as high starch content, excellent palatability, low toxin levels, and reduced breeding costs (1, 7). Consequently, a growing number of livestock enterprises are adopting HMC in their operations. Research by Knowlton et al. (8) demonstrated that HMC exhibits higher ruminal starch digestibility compared to dry corn and steam-flaked corn. Widely adopted across Europe and North America, HMC is now utilized in over 80% of dairy operations within these regions (2, 9). In beef cattle production, Jaconaci et al. (10) conducted a meta-analysis revealing that HMC provides 25.7% higher feeding value than dry corn. Coulson et al. (11) observed that finishing cattle fed HMC exhibited superior feed-to-gain ratios and starch digestibility, accompanied by reduced fecal starch excretion, when compared to those receiving dry corn. In a comparative feeding trial, Stock et al. (12) evaluated three forms of corn (dry-rolled corn, whole-grain HMC, and crushed HMC) in 12-month-old bulls. Their results showed that animals fed crushed HMC had significantly higher daily weight gains and improved feed efficiency. Currently, while HMC is incorporated at variable inclusion rates in beef cattle feeding programs, research quantifying optimal substitution levels remains scarce in the scientific literature. This study investigated the effects of substituting conventional corn with HMC at three inclusion levels on growth performance, rumen fermentation parameters, rumen microbiota diversity, and metabolic profiles in growing Simmental bulls to establish scientific guidelines for optimizing HMC utilization in beef cattle production systems.

## 2 Materials and methods

### 2.1 Study conditions

The experiment was conducted at the Wugongtai Beef Cattle Breeding Cooperative in Hutubi County, Changji Hui Autonomous Prefecture, Xinjiang, from June to October 2024. All animal experiments were approved by the Ethics Review Committee of Xinjiang Agricultural University (Approval No: 20240125).

### 2.2 Materials

The HMC (bale-wrapped, variety Huimin 401) was obtained from Xinjiang Tianrun Co., Ltd. (Shawan, China). Experimental equipment and reagents were sourced as follows: a truck scale (Shanghai Yaohua Truck Scale Co., Ltd., Shanghai, China), a tape measure for body measurements and reagents for VFA analysis (Xinjiang Ziqi Biotechnology Co., Ltd., Urumqi, China). Additionally, 5 mL centrifuge tubes, 2 mL cryogenic vials, and sterile gloves were purchased from Beijing Dingguo Changsheng Biotechnology Co., Ltd. (Beijing, China).

### 2.3 Experimental design

A total of 32 healthy Simmental bulls (approximately 12 months old; initial body weight 243.99 ± 13.52 kg) with a uniform genetic background were randomly assigned to four treatment groups (8 bulls per group). The control group (HMC0) was fed a basal Total Mixed Ration (TMR). The treatment groups received diets in which conventional corn in the basal TMR was replaced with 15%, 30%, or 45% HMC on a dry matter basis, designated as the HMC15, HMC30, and HMC45 groups, respectively. The trial consisted of a 10-day (d) adaptation period followed by a 120 d main experimental period. During the adaptation period, the dietary inclusion level of HMC was gradually increased every two days (0, 15, 30, to 45%) to allow the cattle to acclimate to the diet, management routines, and housing environment prior to the commencement of the main trial.

### 2.4 Feed and management

All experimental cattle were sourced from the same farm, and cattle within the same experimental group were housed together in a shared pen. A Total Mixed Ration (TMR) was provided, with feed delivered at fixed amounts twice daily at 8:00 a.m. and 6:00 p.m. Ad libitum water access was ensured throughout the study period. Standard herd management practices, including routine disease prevention, were maintained under uniform environmental conditions. The diets for all groups were formulated using NDS Pro software; the composition and nutritional profile of the basal TMR are presented in Table 1.

**Table 1:** Experimental basal TMR composition and nutritional level (dry matter basis).

Items	Group			
	HMC0	HMC15	HMC30	HMC45
Ration composition				
Wheat straw/%	20	20	20	20
Whole-plant corn silage/%	30	30	30	30
HMC/%	0	4.5	9	13.5
Conventional corn/%	30	25.5	21	16.5
Wheat bran/%	6.5	6.5	6.5	6.5
Cottonseed meal/%	5	5	5	5
Soybean meal/%	5	5	5	5
NaHCO_3_/%	0.5	0.5	0.5	0.5
Salt/%	0.5	0.5	0.5	0.5
Premix^a^/%	2.5	2.5	2.5	2.5
Total	100	100	100	100
Nutritional level				
CP/%	14.64	14.26	14.47	14.52
EE/%	4.35	4.57	4.46	4.37
NDF/%	44.16	43.69	42.82	42.74
ADF/%	23.63	23.19	22.85	22.56
Net energy for maintenance and fattening (NE_mf_)^b^/MJ/kg	6.28	6.15	6.21	6.24
Ca/%	0.74	0.73	0.73	0.73
P/%	0.37	0.36	0.37	0.36

a The premix provides the following per kilogram of diet: Vitamin A 130,000 IU, Vitamin D₃ 40,000 IU, Vitamin E 1,400 IU, copper (as tribasic copper chloride) 620 mg, iron (as ferrous sulfate) 1,500 mg, manganese (as manganese sulfate) 1,100 mg, zinc (as zinc sulfate) 900 mg, iodine (as calcium iodate) 20–30 mg, selenium 10 mg, cobalt 25 mg, calcium 10%, total phosphorus 1.5%, and sodium chloride 12%.

b The comprehensive net energy of the basal TMR was calculated according to the feeding standard of beef cattle (NY/T 815–2004), while all other nutrient indices were measured values.

### 2.5 Sample collection and measurement

During the experimental period, feed intake was recorded daily by weighing the offered feed prior to morning feeding and measuring the orts at 8:00 a.m. the following day, allowing calculation of average daily feed intake (ADFI) and gain-to-feed ratio (G/F). Body weights were measured after 12-h fasting on days 0, 30, 60, and 120 of the trial to determine ADG. On day 120 of the trial, six experimental cattle per group were randomly selected for sampling 2 h post-morning feeding. The cattle were secured in a restraint chute, and a sterilized flexible silicone tube (disinfected with 75% ethanol and UV irradiation) attached to an oral rumen fluid sampler (Model A1164K, Kelibo Animal Husbandry Technology Co., Ltd., Wuhan, China) was gently inserted into the rumen to a depth of approximately 1.2 m. After 2–3 gentle withdrawals, ~100 mL of rumen fluid was collected, filtered through four layers of sterile gauze within ≤30 s, and immediately measured for pH in triplicate using a calibrated pH meter (Model PHS-3C, Shanghai Yidian Scientific Instruments Co., Ltd., Shanghai, China). The remaining sample was aliquoted into pre-chilled 2 mL cryovials (1.5 mL per tube), flash-frozen in liquid nitrogen within 1 min, and stored at −80 °C for subsequent analyses.


$$\begin{array}{l} \text{ADFI}=(\text{total feed offered}-\text{total orts})/\\ \text{experimental duration}\;(\text{days}) \end{array}$$

$$\begin{array}{l} \mathrm{ADG}=(\text{final body weight}-\text{initial body weight})/\\ \text{experimental days} \end{array}$$

G/F=gain/feed

The cost of gain, trial expenditure, income, and profit were calculated based on the total weight gain, feed intake, and feed cost per experimental bull using the following formulae:


$$\begin{array}{l} \text{Cost of gain}\;(\text{¥}/\mathrm{kg})=\text{feed price}\;(\text{¥}/\mathrm{kg})\times \\ \text{feed conversion ratio}\;(\mathrm{FCR}) \end{array}$$

$$\begin{array}{l} \text{Trial expenditure}\;(\text{¥}/\text{head})=\text{Total Weight Gain}\;(\mathrm{kg}/\text{head})\times \\ \text{Cost of Gain}\;(\text{¥}/\mathrm{kg}) \end{array}$$

$$\begin{array}{l} \text{Trial income}\;(\text{¥}/\text{head})=\text{total weight gain}\;(\mathrm{kg}/\text{head})\times \\ \text{live weight price of beef cattle}\;(\text{¥}/\mathrm{kg}) \end{array}$$

$$\begin{array}{l} \text{Profit}\;(\text{¥}/\text{head})=\text{trial income}\;(\text{¥}/\text{head})—\\ \text{trial expenditure}\;(\text{¥}/\text{head}) \end{array}$$

### 2.6 Measurement of rumen fluid indicators

#### 2.6.1 Determination of rumen fermentation indicators

Ten milliliters of rumen fluid were centrifuged at 15,000 × g for 15 min using a centrifuge (model GT10-1, Beili Centrifuge Co., Ltd., Beijing, China). The supernatant was then collected into a 10 mL test tube and stored for subsequent analysis. The method used by McAuliffe et al. (13) for determining the LA content. According to the method described by Wang et al. (14), the content of VFA was determined: 1 mL of supernatant was transferred to a 1.5 mL centrifuge tube, followed by the addition of 0.5 mL of 10% trichloroacetic acid (Ziqí Biotechnology Co., Ltd., Ürümqi, China) and 0.1 mL of 40 mM 4-methylvaleric acid (Ziqí Biotechnology Co., Ltd., Ürümqi, China). The mixture was vortexed and allowed to stand at room temperature for 30 min. Afterward, it was centrifuged again at 15,000 × g for 15 min. Finally, 1 mL of the resulting supernatant was collected into a screw-capped test tube and analyzed for volatile fatty acids (VFAs) using a gas chromatograph (Agilent Technologies, California, USA). The mixed standard solution was prepared by adding 660 μL of acetic acid (AA), 300 μL of propionic acid (PA), 150 μL of n-butyric acid (BA), 30 μL of isobutyric acid, 35 μL of isovaleric acid, and 40 μL of valeric acid (VA) into a 100 mL volumetric flask, followed by dilution to volume with distilled water. This solution was then used for standard curve construction.

#### 2.6.2 Determination of rumen flora diversity

*DNA extraction*: genomic DNA was extracted from the samples using the CTAB method. The purity and concentration of the DNA were then assessed by agarose gel electrophoresis. An appropriate aliquot of the sample DNA was transferred to a micro centrifuge tube and diluted to 1 ng/μL using nuclease-free water (MetWare Biotechnology Co., Ltd., Wuhan, China).

*PCR amplification*: Using the diluted genomic DNA as template, PCR amplification was performed with barcoded specific primers and Phusion^®^ High-Fidelity PCR Master Mix with GC Buffer (New England Biolabs, USA), which contains a high-efficiency, high-fidelity DNA polymerase, to ensure both amplification efficiency and accuracy (see Table 2).

**Table 2:** Amplified sequence information.

Items	Primer sequences (5′—3′)	Annealing temperature (°C)
16S V4	515F: GTGCCAGCMGCCGCGGTAA	50
	806R: GGACTACHVGGGTWTCTAAT	

*Pooling and purification of PCR products*: PCR products were subjected to electrophoresis analysis using a 2% agarose gel (Thermo Fisher Scientific, Shanghai, China). PCR products that passed quality control were purified using magnetic bead purification, quantified via microplate spectrophotometry, and equal volumes were mixed based on their concentrations. The pooled mixture was thoroughly homogenized before being analyzed by 2% agarose gel electrophoresis. Target bands were subsequently isolated using the Qiagen Gel Extraction Kit following the manufacturer’s protocol.

*Library preparation and sequencing*: Library construction was performed using the TruSeq^®^ DNA PCR-Free Sample Preparation Kit (Illumina, San Diego, California). The quality-qualified libraries were quantified by both Qubit fluorometry and quantitative PCR before being subjected to sequencing on the NovaSeq 6,000 platform (Illumina, San Diego, California).

*Sequencing data quality control and ASV processing*: Based on the barcode sequences and PCR amplification primer sequences, the raw sequencing data were demultiplexed to extract individual sample datasets. High-quality reads were obtained by filtering the original reads using fastp. Subsequently, FLASH was employed to merge paired-end reads into clean tags (high-quality tag sequences). Chimeric sequences were identified and removed through comparison with a reference species annotation database using vsearch, resulting in the generation of effective tags. These effective tags were further processed using the DADA2 method for denoising and filtering, equivalent to clustering at 100% similarity, to generate Amplicon Sequence Variants (ASVs). The ASVs were then annotated with taxonomic information using the classify-sklearn algorithm implemented in QIIME2, elucidating the compositional structure of microbial communities within the samples. Additionally, an in-depth analysis of species diversity and relative abundance was performed. Alpha diversity indices (e.g., Chao1 and Simpson) and Beta diversity analyses were calculated using qiime2 software. Visualization tools such as bar charts and correlation heatmaps were utilized to compare the richness and evenness of ASVs across different samples.

### 2.7 Metabolomics

Based on the analysis of growth performance in young Simmental bulls, rumen metabolic profiles were compared between the optimal HMC substitution group and the control group to identify key metabolic differences. These findings contribute to elucidating critical metabolic pathways within the rumen.

#### 2.7.1 Sample preparation

The sample stored at −80 °C refrigerator was thawed on ice and vortexed for 10 s. A 150 μL extract solution (ACN: Methanol = 1:4, V/V) containing internal standard was added into 50 mg sample. Then the sample was vortex for 3 min and centrifuged at 12,000 rpm for 10 min (4 °C). A 150 μL aliquots of the supernatant was colleted and placed in −20 °C for 30 min, and then centrifuged at 12,000 rpm for 3 min (4 °C). 120 μL aliquots of supernatant were transferred for LC–MS analysis.

#### 2.7.2 HPLC conditions

All samples were for two LC/MS methods. One aliquot was analyzed using positive ion conditions and was eluted from T3 column (Waters ACQUITY Premier HSS T3 Column 1.8 μm, 2.1 mm*100 mm) using 0.1% formic acid(Thermo Fisher Scientific, Shanghai, China) in water as solvent A and 0.1% formic acid(Thermo Fisher Scientific, Shanghai, China) in acetonitrile as solvent B in the following gradient: 5% to 20% in 2 min, increased to 60% in the following 3 min, increased to 99% in 1 min and held for 1.5 min, then come back to 5% mobile phase B witnin 0.1 min, held for 2.4 min. The analytical conditions were as follows, column temperature, 40 °C; flow rate, 0.4 mL/min; injection volume, 4 μL; Another aliquot was using negative ion conditions and was the same as the elution gradient of positive mode.

#### 2.7.3 MS conditions (QE)

All the methods alternated between full scan MS and data dependent MSn scans using dynamic exclusion. MS analyses were carried out using electrospray ionization in the positive ion mode and negative ion mode using full scan analysis over m/z 75–1,000 at 35,000 resolution. Additional MS settings are: ion spray voltage, 3.5 KV or 3.2 KV in positive or negative modes, respectively; Sheath gas (Arb), 30; Aux gas, 5; Ion transfer tube temperature, 320 °C; Vaporizer temperature, 300 °C; Collision energy, 30, 40, and 50 V; Signal Intensity Threshold, 1*e6 cps; Top N vs. Top speed, 10; Exclusion duration, 3 s.

#### 2.7.4 Methods of analysis

*PCA*: Unsupervised PCA (principal component analysis) was performed by statistics function prcomp within R.^1^ The data was unit variance scaled before unsupervised PCA.

*Hierarchical Cluster Analysis and Pearson Correlation Coefficients*: The HCA (hierarchical cluster analysis) results of samples and metabolites were presented as heatmaps with dendrograms, while Pearson correlation coefficients (PCC) between samples were calculated by the cor function in R and presented as only heatmaps. Both HCA and PCC were carried out by R package ComplexHeatmap. For HCA, normalized signal intensities of metabolites (unit variance scaling) are visualized as a color spectrum.

*Differential metabolites selected*: For two-group analysis, differential metabolites were determined by VIP (VIP > 1) and *p*-value (*p*-value < 0.05, Student’s t test). For multi-group analysis, differential metabolites were determined by VIP (VIP > 1) and *p*-value (*p*-value < 0.05, ANOVA). VIP values were extracted from Orthogonal Partial Least Squares Discriminant Analysis (OPLS-DA) result, which also contain score plots and permutation plots, was generated using R package MetaboAnalystR. The data was log transform (log_2_) and mean centering before OPLS-DA. In order to avoid overfitting, a permutation test (200 permutations) was performed.

*KEGG annotation and enrichment analysis*: Identified metabolites were annotated using KEGG Compound database,^2^ annotated metabolites were then mapped to KEGG Pathway database.^3^

### 2.8 Statistical analysis

Data were processed and organized using Excel 2016 (Microsoft Corporation, USA), followed by one-way ANOVA analysis. The homogeneity of variance was assessed using SPSS Statistics 20.0 (IBM Corporation, USA). Results are presented as the mean ± standard deviation. Differences were considered statistically significant at *p* < 0.05, highly significant at *p* < 0.01, and non-significant at *p* > 0.05.

## 3 Results

### 3.1 Effects of feeding HMC on growth performance in young Simmental bulls

As shown in Table 3, compared with the HMC0 group, the body weight of experimental cattle in the HMC30 and HMC45 groups increased significantly by 7.29 and 8.63%, respectively, on day 120 (*p* < 0.05). The ADG of the HMC15, HMC30, and HMC45 groups was significantly higher than that of the HMC0 group during the 0–30 d, 60–120 d, and 0–120 d periods (*p* < 0.05), and was also significantly higher during the 30–60 d period (*p* < 0.01). In the 30–60 d and 60–120 d periods, the ADFI of the HMC45 group was significantly higher than that of the control group (*p* < 0.05). During the 0–30 d, 30–60 d, and 60–120 d periods, the G/F of the HMC15, HMC30, and HMC45 groups was significantly higher than that of the HMC0 group (*p* < 0.05). Additionally, during the 0–120 d period, the G/F of the HMC45 group was significantly higher than that of the HMC0 group (*p* < 0.05). Based on the final body weight, ADG, ADFI, and G/F across all treatment groups, the HMC45 group was identified as the optimal high-moisture corn substitution group.

**Table 3:** Effects of feeding HMC on the growth performance of young Simmental bulls.

Items	HMC0	HMC15	HMC30	HMC45	SEM	*P*-value
Weight (kg)						
0 d	241.50	240.46	247.08	246.92	2.76	0.78
30 d	267.83	270.38	277.54	278.29	2.91	0.052
60 d	296.17	305.42	313.08	314.17	3.31	0.19
120 d	356.00^b^	372.42^a,b^	381.96^a^	386.71^a^	4.29	0.045
ADG (kg/d)						
0–30 d	0.88^B^	0.99^A^	1.02^A^	1.05^A^	0.02	0.01
30–60 d	0.94^b^	1.17^a^	1.19^a^	1.20^a^	0.04	0.03
60–120 d	1.00^B^	1.12^A^	1.15^A^	1.21^A^	0.03	0.01
0–120 d	0.95^B^	1.10^A^	1.12^A^	1.17^A^	0.02	<0.01
ADFI (kg/d)						
0–30 d	8.42	8.88	9.08	9.20	0.97	0.08
30–60 d	9.21^b^	10.18^a,b^	10.40^a,b^	10.46^a^	0.99	0.046
60–120 d	10.40^b^	11.27^a,b^	11.55^a,b^	11.81^a^	1.38	0.048
0–120 d	9.34	10.11	10.34	10.49	1.43	0.051
G/F						
0–30 d	0.104^b^	0.112^a^	0.112^a^	0.114^a^	0.005	0.035
30–60 d	0.103^b^	0.115^a^	0.114^a^	0.114^a^	0.008	0.023
60–120 d	0.092^b^	0.098^a^	0.097^a^	0.099^a^	0.004	0.049
0–120 d	0.102^b^	0.109^a,b^	0.109^a,b^	0.111^a^	0.005	0.038

Within the same row, different lowercase letters denote significant differences (*p* < 0.05), while different uppercase letters indicate highly significant differences (*p* < 0.01). No letter or identical letters indicate no significant difference (*p* > 0.05). Unless otherwise specified, subsequent tables follow the same labeling convention.

### 3.2 Effects of HMC on economic benefits in young Simmental bulls

As shown in Table 4, the profit per experimental bull increased progressively with higher dietary HMC inclusion levels. All HMC-supplemented groups yielded greater profits than the HMC0, with the HMC45 group achieving the highest profit, which was ¥491.47 greater per head than the HMC0 group.

**Table 4:** Effects of feeding HMC on economic benefits of young Simmental bulls.

Items	HMC0	HMC15	HMC30	HMC45
Cost of gain/(¥ per kg)	1.52	1.50	1.48	1.46
Weight gain cost/(¥ per kg)	14.85	13.78	13.63	13.17
Trial expenditure /(¥ per head)	1700.77	1818.56	1838.42	1841.57
Trial income/(¥ per head)	2862.50	3298.98	3371.88	3494.78
Profit/(¥ per head)	1161.73	1480.42	1533.45	1653.20

The unit prices in the diet were ¥1.90 kg^−1^ for HMC and ¥2.50 kg^−1^ for cracked corn, with identical other ingredients. For the economic analysis, medical costs, labor costs, and income from cattle manure were considered to offset each other, resulting in a net value of zero. Feed cost was calculated based on the unit price per kilogram of feed and the actual feed intake. The selling price of beef cattle was set at ¥25.00 kg^−1^.

### 3.3 Effects of HMC on rumen fermentation in young Simmental bulls

As presented in Table 5, although no significant differences (*p* > 0.05) were observed in pH values among treatment groups, a decreasing trend was evident with increasing dietary inclusion levels of HMC. The LA content in the HMC0 and HMC15 groups was significantly lower than that in the HMC30 and HMC45 groups, while the AA content was significantly higher than that in the HMC30 and HMC45 groups (*p* < 0.05). Additionally, the total VFA concentration in the HMC0, HMC15, and HMC30 groups was significantly higher than that in the HMC45 group (*p* < 0.05).

**Table 5:** Effects of HMC on rumen fermentation in young Simmental bulls.

Items	HMC0	HMC15	HMC30	HMC45	SEM	*P*-value
pH	6.87	6.85	6.77	6.59	0.06	0.08
LA (mmol/L)	23.51^b^	24.85^b^	33.24^a^	36.50^a^	0.57	0.02
AA (mmol/L)	48.62^a^	49.72^a^	42.88^b^	38.36^b^	1.87	0.04
PA (mmol/L)	10.92	9.86	9.63	9.47	0.29	0.30
BA (mmol/L)	5.77	5.41	5.44	5.23	0.23	0.77
VA (mmol/L)	0.36	0.36	0.36	0.34	0.01	0.87
Total VFA (mmol/L)	66.59^a^	66.44^a^	59.45^a^	54.42^b^	3.28	0.04

### 3.4 Effects of HMCn on rumen microbial diversity in young Simmental bulls

#### 3.4.1 Alpha diversity analysis of ruminal microbial

The comparative analysis of rumen microbial alpha diversity indices among treatment groups (Table 6) showed that the HMC45 group had significantly lower Chao1 index than HMC15 and HMC30 groups (*p* < 0.05). The Simpson index in HMC0 was significantly lower than all other groups (*p* < 0.05). Furthermore, while the Shannon index of HMC45 was significantly lower than that of HMC15 and HMC30 groups, it remained significantly higher than HMC0 group (*p* < 0.05).

**Table 6:** Effects of HMC on ruminal microbial alpha diversity in young Simmental bulls.

Items	HMC0	HMC15	HMC30	HMC45	SEM	*P*-value
Chao1 index	931.11^ab^	1002.74^a^	1009.64^a^	882.47^b^	19.95	0.04
Simpson index	0.98^b^	1.00^a^	1.00^a^	0.99^a^	0.02	0.03
Shannon index	5.54^c^	6.03^a^	6.092^a^	5.901^b^	0.05	0.02

#### 3.4.2 Ruminal microbiota beta diversity

**Figure 1:**
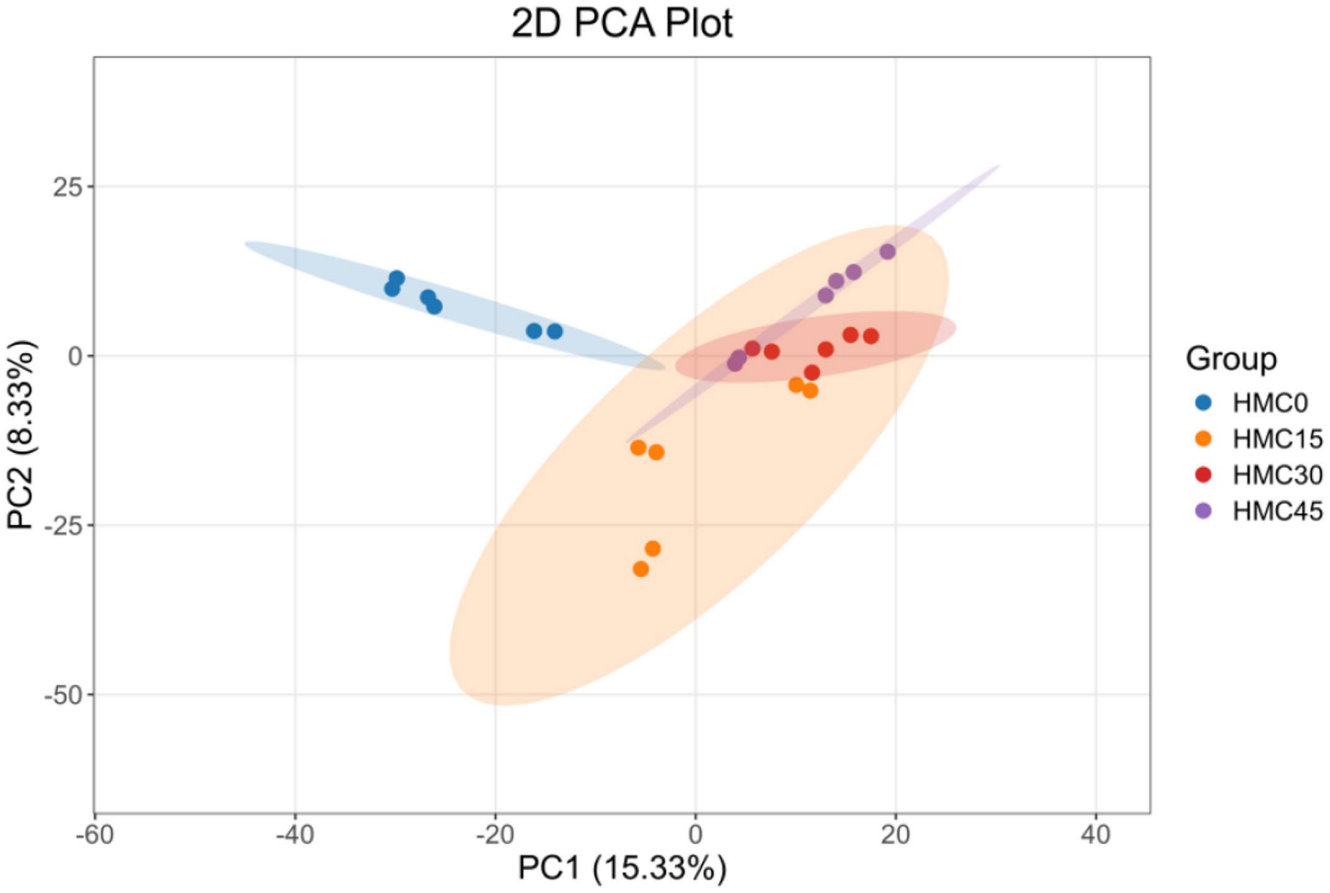
PCA of ruminal microbial communities based on OTUs. The *x*-axis represents the first principal component with the percentage indicating its contribution value to sample variation while the *y*-axis shows the second principal component with its corresponding percentage contribution value. Each point in the figure represents an individual sample with samples from the same group displayed in identical colors.

Principal component analysis of rumen microbiota at the OTU level in young Simmental bulls (Figure 1) revealed that PC1 and PC2 accounted for 15.33 and 8.33% of the variance respectively, with good clustering observed among samples within each group. The microbial communities showed overlapping distributions between HMC15 and HMC45 groups as well as between HMC15 and HMC30 groups, while the HMC0 group remained distinctly separated from the other three treatment groups in bacterial community composition.

#### 3.4.3 Phylum-level composition and relative abundance of ruminal microbial communities

The phylum-level distribution of rumen microbiota across treatment groups is presented in Table 7. The data show that Bacteroidota and Firmicutes collectively accounted for over 85% of relative abundance, dominating the microbial community. Among these, the total relative abundance of dominant phyla in the HMC45 group was higher than that in the other groups. Notably, the Bacteroidota abundance in HMC0 was significantly higher than in HMC15 and HMC30 (*p* < 0.01), while Firmicutes abundance in HMC0 was markedly lower than in HMC30 (*p* < 0.01) and significantly lower than in HMC15 and HMC45 (*p* < 0.05). Additionally, Verrucomicrobiota abundance in HMC45 was substantially reduced compared to HMC0 (*p* < 0.01) and HMC15 (*p* < 0.05).

**Table 7:** Distribution of rumen microbial at phylum level %.

Items	HMC0	HMC15	HMC30	HMC45	SEM	*P*-value
Bacteroidota	49.25^A^	41.36^B^	41.26^B^	44.61^A,B^	0.86	0.01
Firmicutes	38.24^B,b^	44.58^A,B,a^	46.85^A,a^	43.92^A,B,a^	0.90	<0.01
Actinobacteriota	3.68	3.40	3.00	3.28	0.20	0.73
Fibrobacterota	3.22	4.51	3.75	3.87	0.28	0.48
Verrucomicrobiota	2.04^A^	1.90^A,B,a^	1.27^A,B^	0.93^B,b^	0.14	0.01
Proteobacteria	1.87	0.82	0.60	0.66	0.20	0.07
Desulfobacterota	0.55	0.81	1.11	0.88	0.06	<0.01
Spirochaetota	0.31	0.76	0.68	0.66	0.07	0.09

#### 3.4.4 Genus-level composition and relative abundance of ruminal microbiota

The genus-level distribution of rumen microbiota across treatment groups is presented in Table 8. *Prevotella*, *Fibrobacter*, *unidentified_Bacteroidales*, and *Ruminococcus* showed higher relative abundances among all genera. Specifically, the combined relative abundance of these four bacterial genera in the HMC45 group was higher than in the other groups. Notably, *Desulfovibrio* and *Succiniclasticum* abundances in HMC0 were significantly lower than in HMC30 (*p* < 0.01), while *Acetitomaculum* in HMC0 was reduced compared to HMC45 (*p* < 0.05). Similarly, HMC0 had lower *unidentified_Bacteroidales* versus HMC30 (*p* < 0.05) but higher *Olsenella* (*p* < 0.05), with *Succiniclasticum* being lower than both HMC30 and HMC45 (*p* < 0.05). Furthermore, *Prevotella* in HMC30 was markedly lower than in HMC45 (*p* < 0.01), whereas its *Succiniclasticum* abundance exceeded HMC15 (*p* < 0.05).

**Table 8:** Distribution of rumen microbial at genus level %.

Items	HMC0	HMC15	HMC30	HMC45	SEM	*P*-value
*Prevotella*	12.73^A,B^	11.73^A,B^	10.17^B^	15.61^A^	0.62	0.01
*Desulfovibrio*	0.54^B^	0.76^A,B^	1.10^A^	0.86^A,B^	0.06	<0.01
*Fibrobacter*	3.17	4.46	3.69	3.81	0.28	0.48
*unidentified_Bacteroidales*	2.06^b^	3.03^a,b^	4.43^a^	4.12^a,b^	0.32	0.03
*Ruminococcus*	2.71	3.32	3.40	3.73	0.17	0.18
*Olsenella*	1.69^a^	1.16^a,b^	0.43^b^	0.51^b^	0.18	0.02
*Succiniclasticum*	1.44^B,c^	1.78^A,B,bc^	2.38^A,a^	2.06^A,B,a,b^	0.09	<0.01
*Acetitomaculum*	1.30^b^	1.97^a,b^	1.89^a,b^	2.31^a^	0.12	0.02

### 3.5 Effects of HMC on rumen metabolism in young Simmental bulls

#### 3.5.1 OPLS-DA results of metabolites

**Figure 2:**
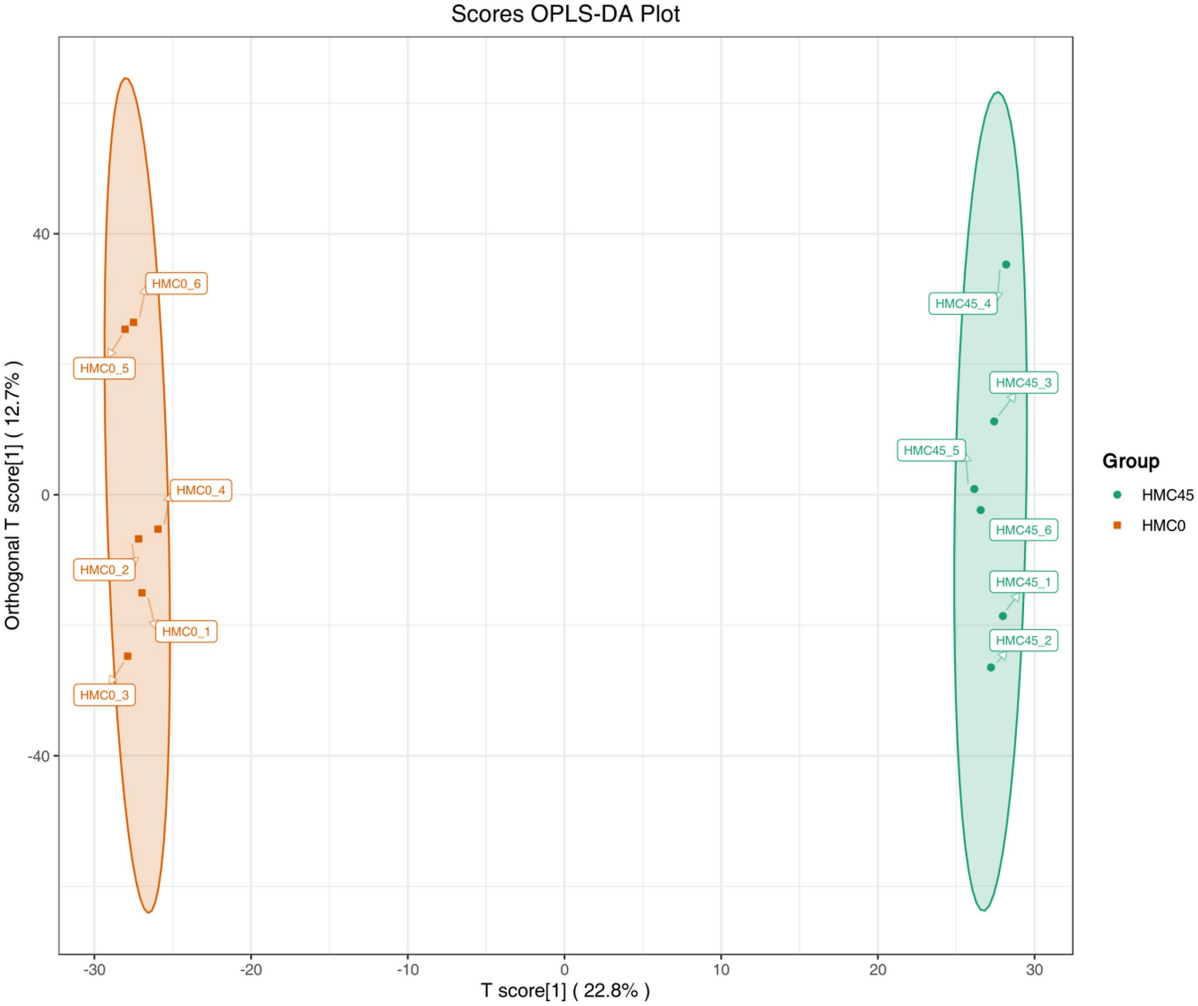
OPLS-DA results of rumen metabolites between HMC45 and HMC0 groups. The *x*-axis represents the predictive component, where the direction indicates between-group variation, while the *y*-axis shows the orthogonal component reflecting within-group variation, with percentages denoting each component’s explained variance. Each point corresponds to an individual sample, where samples from the same group share identical colors as labeled by “Group”.

By comparing the effects of feeding different levels of HMC on the production performance of experimental cattle, this study selected a control group (HMC0, i.e., the group without HMC supplementation) and an optimal feeding level group (HMC45, i.e., the group supplemented with 45% HMC) to conduct rumen fluid metabolomics analysis, aiming to investigate the changes in metabolites and metabolic pathways in the bodies of these two groups of experimental cattle. The OPLS-DA results demonstrated clear separation between these two groups, with all samples falling within the 95% confidence interval (Figure 2).

#### 3.5.2 Differential metabolite analysis

**Figure 3:**
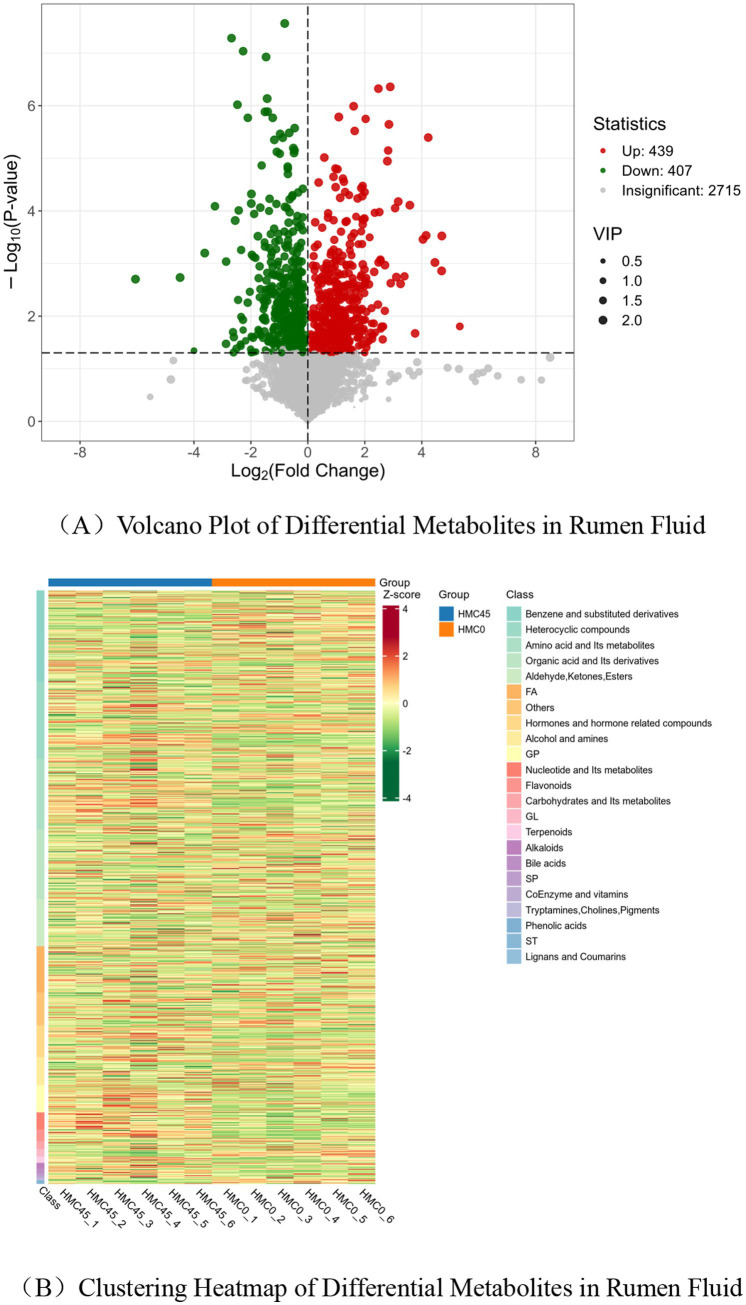
Differential metabolite analysis of HMC45 vs. HMC0. **(A)** Each point in the volcano plot represents a metabolite: green indicates downregulated differential metabolites, red indicates upregulated differential metabolites, and gray denotes metabolites detected but without significant differences. The *x*-axis shows the log_2_-transformed fold change (FC) of relative abundance between the two groups–larger absolute values indicate greater abundance differences. Under the screening criteria (VIP + FC + *p*-value), the *y*-axis represents the significance level [−log10(*p*-value)], and dot size corresponds to the VIP value. **(B)** The heatmap displays sample names (horizontal) and differential metabolite information (vertical), with “Group” indicating the experimental groupings. Colors reflect normalized relative abundance values (red = high abundance, green = low abundance). Heatmap_class, metabolite-subclassified heatmap; “Class” denotes the primary classification of metabolites.

Volcano plot analysis of differential metabolites in rumen fluid identified a total of 2,715 metabolites under positive and negative ion modes. Using the screening criteria of |FC| > 1, VIP > 1.0, and *p* < 0.05, 846 differential metabolites were annotated, among which 439 were upregulated and 407 were downregulated (Figure 3A). The differential metabolites exhibited clear clustering between groups, as visualized in the heatmap (Figure 3B).

**Figure 4:**
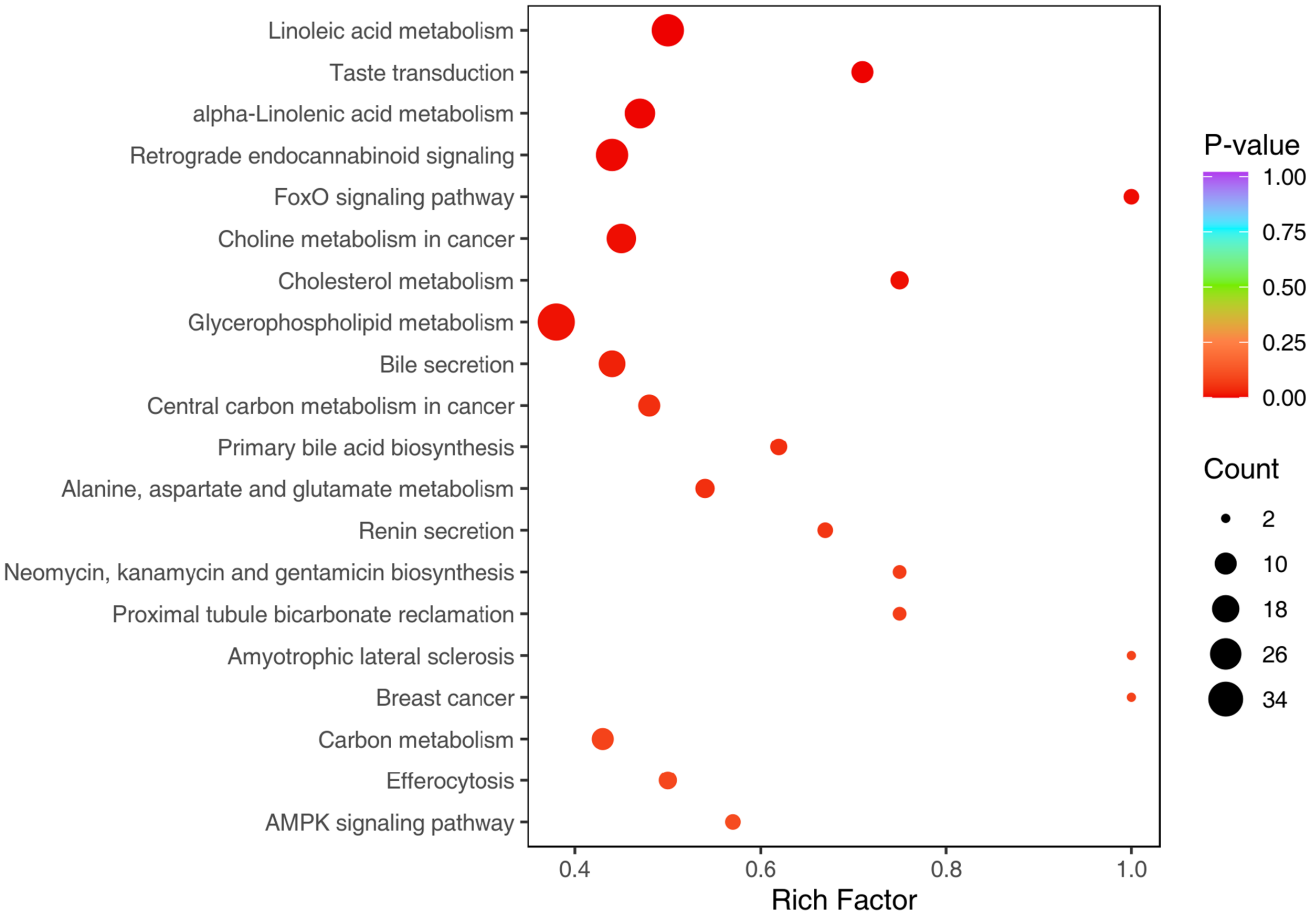
KEGG pathway enrichment bubble chart of differentially metabolized substances in HMC45 vs. HMC0. The horizontal coordinate indicates the corresponding rich factor for each pathway, and the vertical coordinate is the pathway name (sorted by *p*-value). The color of the dots reflects the size of the *p*-value, the redder it is, the more significant the enrichment. The color of the dots reflects the size of *p*-value, the redder it is, the more significant the enrichment is, and the size of the dots represents the number of different metabolites enriched, the closer the *p*-value is to 0, the more significant the enrichment is.

According to the KEGG enrichment analysis results of the differential metabolites (Figure 4), the differential metabolites in the rumen fluid of the two groups of experimental bulls were mainly enriched in 20 entries. Among these, 12 entries had *p*-values less than 0.05. These entries were: linoleic acid metabolism, taste transduction, alpha-linolenic acid metabolism, FoxO signaling pathway, eholine metabolism in cancer, cholesterol metabolism, glycerophospholipid metabolism, bile secretion, retrograde endocannabinoid signaling, central carbon metabolism in cancer, alanine, aspartate, and glutamate metabolism, and primary bile acid biosynthesis.

A total of 52 differentially expressed metabolites were screened from 12 metabolic pathways, of which 34 showed an upregulation trend and 18 showed a downregulation trend (Table 9). Among these 12 metabolic pathways, there are recurring differentially expressed metabolites: 1,2-dioleoyl-sn-glycero-3-phosphocholine, diooleoylphosphatidylcholine, and PC (18,3/18:3) were significantly downregulated (*p* < 0.01) in the following five pathways: glycerophospholipid metabolism, choline metabolism in cancer, linoleic acid metabolism, retrograde endocannabinoid signaling, and alpha-linolenic acid metabolism. In contrast, LPC (16,1/0:0) showed a highly significant upregulation (*p* < 0.01) in these five pathways. Taurocholic acid and taurochenodeoxycholic acid showed extremely significant upregulation (*p* < 0.01) in three pathways: bile secretion, primary bile acid biosynthesis, and cholesterol metabolism. L-glutamic acid is involved in reverse endogenous cannabinoid signaling, central carbon metabolism in cancer, alanine, aspartate, and glutamate metabolism, taste transduction, and the FoxO signaling pathway. Succinic acid showed extremely significant upregulation (*p* < 0.01) in two pathways in cancer: central carbon metabolism and alanine, aspartate, and glutamate metabolism. *γ*-aminobutyric acid (GABA) showed extremely significant upregulation (*p* < 0.01) in two pathways: alanine, aspartic acid, and glutamic acid metabolism, and taste conduction. Adenosine monophosphate (AMP) and adenosine 5′-diphosphate (AMP) showed extremely significant upregulation (*p* < 0.01) in the taste conduction and FoxO signaling pathways, respectively. Furthermore, the metabolic differences enriched in the HMC45 group were involved to varying degrees in lipid metabolism, protein and amino acid metabolism, and other metabolic pathways.

**Table 9:** Differential metabolic pathways and metabolites between HMC45 and HMC0 rumen liquids.

Metabolic pathway	*P*-value	Differential metabolites	VIP	*P*-value
Glycerophospholipid metabolism	0.0099	1,2-Dioleoyl-sn-glycero-3-phosphocholine ↓	2.036	0.0006
		Dioleoylphosphatidylcholine ↓	2.057	0.0018
		LPC(16:1/0:0) ↑	1.9164	0.0013
		LPE(16:0/0:0) ↑	1.9725	<0.0001
		Diethanolamine ↑	1.9110	0.0001
		5,8,11,14-Eicosatetraenoic acid ↑	1.9303	<0.0001
		PC(18:3/18:3) ↓	1.8203	0.0252
Bile secretion	0.0254	Taurocholic acid(TCA) ↑	2.0242	<0.0001
		Taurochenodeoxycholic acid(TCDCA) ↑	2.0663	<0.0001
		6-trans leukotriene B4 ↓	1.7244	0.0010
		Cyclic guanosine monophosphate ↑	1.6049	0.0039
Choline metabolism in cancer	0.0074	1,2-Dioleoyl-sn-glycero-3-phosphocholine ↓	2.036	0.0006
		Dioleoylphosphatidylcholine ↓	2.0574	0.0018
		LPC(16:1/0:0) ↑	1.9164	0.0013
		PC(18:3/18:3) ↓	1.8203	0.0252
		Arachidonoyl PAF C-16 ↑	1.771	0.0065
Linoleic acid metabolism	0.0003	1,2-Dioleoyl-sn-glycero-3-phosphocholine ↓	2.036	0.0006
		Dioleoylphosphatidylcholine ↓	2.0574	0.0018
		LPC(16:1/0:0) ↑	1.9164	0.0013
		PC(18:3/18:3) ↓	1.8203	0.0252
Primary bile acid biosynthesis	0.0443	Taurocholic acid(TCA) ↑	2.0242	<0.0001
		Taurochenodeoxycholic acid(TCDCA) ↑	2.0663	<0.0001
Retrograde endocannabinoid signaling	0.0040	1,2-Dioleoyl-sn-glycero-3-phosphocholine ↓	2.036	0.0006
		Dioleoylphosphatidylcholine ↓	2.0574	0.0018
		LPC(16:1/0:0) ↑	1.9164	0.0013
		L-Glutamic acid ↑	1.9589	0.0002
		PC(18:3/18:3) ↓	1.8203	0.0252
Alpha-linolenic acid metabolism	0.0024	1,2-Dioleoyl-sn-glycero-3-phosphocholine ↓	2.036	0.0006
		Dioleoylphosphatidylcholine ↓	2.0574	0.0018
		LPC(16:1/0:0) ↑	1.9164	0.0013
		PC(18:3/18:3) ↓	1.8203	0.0252
Cholesterol metabolism	0.0078	Taurocholic acid(TCA) ↑	2.0242	<0.0001
		Taurochenodeoxycholic acid(TCDCA) ↑	2.0663	<0.0001
		Glycocholic acid ↓	1.5303	0.0062
		Cholesterol ↓	1.5005	0.0178
Central carbon metabolism in cancer	0.0438	L-Glutamic acid ↑	1.9589	0.0002
		Succinic acid ↑	1.9888	0.0003
		Asparagine ↓	1.6988	0.0029
		Malic acid ↑	1.6836	0.0047
		L-Phenylalanine ↑	1.6632	0.0211
Alanine, aspartate and glutamate metabolism	0.0444	L-Glutamic acid ↑	1.9589	0.0002
		Succinic acid ↑	1.9888	0.0003
		γ-Aminobutyric acid(GABA) ↑	1.8199	0.0055
		Ureidosuccinic acid ↑	1.7626	0.0157
Taste transduction	0.0009	L-Glutamic acid ↑	1.9589	0.0002
		Saccharin ↑	1.9584	<0.0001
		γ-Aminobutyric Acid(GABA) ↑	1.8199	0.0055
		Adenosine monophosphate(AMP) ↑	1.7688	0.0016
		Adenosine 5’-diphosphate(ADP) ↑	1.7604	0.0191
FoxO signaling pathway	0.0062	Adenosine monophosphate(AMP) ↑	1.7688	0.0016
		L-Glutamic acid ↑	1.9589	0.0002
		Adenosine 5’-diphosphate(ADP) ↑	1.7604	0.0191

P denotes the significance test *p*-value, where 0.01 < *p* < 0.05 means that the result is statistically significant and *p* < 0.01 means that the result is highly statistically significant. ↑indicates that the differential metabolite is up-regulated, ↓indicates that the differential metabolite is down-regulated. VIP indicates that the calculation of OPLS-DA is proportional to the contribution of metabolites. LPC indicates lysophosphatidylcholine Lysophosphatidylcholine, LPE indicates lysophosphatidylethanolamine, PC indicates phosphatidylcholine.

## 4 Discussion

### 4.1 Effects of feeding HMC on growth performance in young Simmental bulls

The results of this experiment indicate that replacing conventional crushed corn with HMC in the diet fed to young Simmental bulls can improve their growth performance, with the optimal replacement level being 45% HMC. As the HMC replacement level gradually increased, the final body weight, ADG, ADFI, and G/F of the experimental cattle all showed an upward trend. Stock et al. (12) found that the castrated bull group fed crushed HMC had higher average daily weight gain and feed conversion rates than the group fed dry corn, a finding that was consistent with the conclusions of our study. Although several studies on beef cattle fattening have found that HMC does not significantly affect the final weight and daily weight gain of test cattle, its feed conversion rate is higher than that of the control group (15, 16). This may be due to the ensiling characteristics of HMC. Although HMC has a high moisture content, the ensiling process reduces the loss of nutrients in corn kernels and disrupts the starch-protein complex structure in corn (17, 18), thereby maximizing the absorption and utilization of nutrients by the rumen after cattle consumption and improving feed digestion efficiency. Therefore, in production practice, if HMC can be reasonably applied to the basic diet of beef cattle, it is expected to improve the production performance and feed conversion rate of beef cattle, thereby reducing feeding costs.

### 4.2 Effects of feeding HMC on rumen fermentation in young Simmental bulls

Changes in rumen pH directly affect the production of VFA. When the rumen pH is between 6.5 and 6.8, the concentration of VFA reaches its optimal level; if the pH is too low, it increases the risk of acidosis and rumen metabolic diseases (19, 20). In the present study, ruminal pH exhibited a decreasing trend with increasing dietary inclusion of HMC. This finding aligns with previous research; for instance, Peng et al. (21) conducted a 48-h rumen *in vitro* fermentation experiment using conventional corn and HMC in different proportions and found that as the proportion of HMC increased, the rumen pH decreased. Similarly, Coulson et al. (11) reported lower rumen pH in bulls fed ROLL HMC compared to those fed dry corn. This consistent phenomenon can be attributed to the higher ruminal starch digestibility of HMC compared to conventional cracked corn (8). The rapid fermentation of this highly digestible starch in the rumen explains the concomitant decrease in pH with greater HMC inclusion.

VFA, including acetate, propionate, and butyrate, are the primary products of rumen fermentation and serve as a major energy source for ruminants (22). The concentration of VFA reflects the metabolic activity of rumen microbes digesting dietary carbohydrates (23), and upon absorption through the rumen wall, they enter the bloodstream to supply energy or support fat synthesis (24). In the study, we observed that when the HMC substitution level exceeded 30%, ruminal AA concentration decreased significantly, while LA increased. The HMC45 group exhibited the lowest total VFA content among all groups. We speculate that this change is caused by the high-digestibility starch in HMC, which leads to a fundamental alteration in the rumen fermentation pattern. The rapid fermentation likely caused the microbial community to shift from a “fiber-fermenting type” to a “starch-rapid-fermenting type,” which suppresses fibrolytic bacteria and reduces absolute AA production. Concurrently, the accumulation of LA, which failed to be further converted into VFA (primarily PA), contributed to the overall decline in total VFA yield. This *in vivo* dynamic, particularly the accumulation of LA and its impact on total VFA, may not be fully captured in closed, short-term batch-culture *in vitro* systems [e.g., 48-h fermentation (21)], which can explain the apparent discrepancy with studies reporting higher VFA at high HMC levels in vitro.

### 4.3 Effects of feeding HMC on ruminal microbiota diversity in young Simmental bulls

The rumen microbial community is shaped by its environment, and beneficial shifts can enhance nutrient absorption and production performance in ruminants (25). In this study, the HMC45 group exhibited the lowest Chao1 index but higher Shannon and Simpson indices than the HMC0 group, indicating that high HMC inclusion reduced species richness while increasing community evenness and overall diversity. This shift likely results from dietary selection pressure: the rapid fermentation and lower pH induced by HMC may inhibit pH-sensitive fibrolytic bacteria, reducing total species, while simultaneously promoting the proliferation of acid-tolerant and amylolytic bacteria by providing readily fermentable starch. This leads to a more functionally adapted and structurally balanced microbial ecosystem. Our findings show both consonance and divergence with previous studies. The decrease in richness (Chao1) aligns with Liu et al. (26), who reported reduced diversity with acidogenic starch, confirming the inhibitory effect of low pH. Conversely, while the increased diversity (Shannon, Simpson) matches Peng et al. (27) at 60 days, the contrasting richness may reflect sampling time. Our longer-term exposure (120 days) might represent a later successional stage where prolonged acidity continuously filters species, illustrating a dynamic progression from initial microbial proliferation to stable environmental selection.

In ruminants, the phyla Bacteroidota and Firmicutes are the dominant rumen microbiota; Bacteroidota primarily degrades diverse carbohydrates and enhances host immunity and gut homeostasis, whereas Firmicutes mainly decomposes fiber degradation, producing key metabolites like butyrate and propionate that are crucial for gut health (28–32). In the present study, Bacteroidota and Firmicutes were the dominant phyla, with HMC45 exhibiting a Bacteroidota abundance lower than HMC0 but higher than HMC15 and HMC30, and a Firmicutes abundance higher than HMC0 but lower than the other treatment groups. This specific profile suggests that a 45% HMC substitution might favor a microbial community capable of more efficient carbohydrate breakdown compared to lower substitution levels, alongside maintaining a higher fibrolytic capacity than the conventional corn diet, which could potentially enhance overall nutrient degradation and absorption across the rumen wall. Conversely, the relative abundance of Verrucomicrobiota decreased with increasing HMC inclusion. Given its putative association with gut immunity (33), this decline might indicate a heightened risk of subacute acidosis, which could subsequently suppress immune-related microbial populations. However, within the context of this study, the elevated feed intake and average daily gain, coupled with rumen pH values that remained within a safe range, suggest that the decreased Verrucomicrobiota abundance did not translate into overt health impairments under the current experimental conditions.

Further research has found that at the rumen level, the dominant bacterial group is *Prevotella*, which plays a crucial role in maintaining the balance between health and disease in the body (34). The composition of the diet affects *Prevotella*, and fermentable feed with a high degradation rate of carbohydrates is an important source of nutrients for *Prevotella* (35). Our study found that the relative abundance of *Prevotella* in the HMC45 group (45% high-moisture corn replacement group) was higher than that in the other groups. As a fermented feed, HMC, when substituted at a 45% level, provides the rumen microorganisms with a more abundant and readily fermentable starch substrate, thereby significantly enhancing the overall fermentation efficiency of non-fiber carbohydrates. The unidentified Bacteroidales genus belongs to the Bacteroidota phylum. We speculate that its function is similar to that of the Bacteroidota phylum in the rumen, with the ability to degrade carbohydrates. *Succiniclasticum* can break down succinic acid into propionic acid, which is super important for keeping the rumen environment stable (36). *Desulfovibrio* can ferment lactic acid, and how much of it is in the rumen is closely tied to the rumen fermentation process (37). *Acetitomaculum* may play a role in rumen fermentation, converting lactic acid into acetic acid (38). In our study, the relative abundance of unidentified Bacteroidota, *Succinibacter*, *Desulfovibrio*, and *Acetobacter genera* in the rumen of experimental cattle fed HMC was higher than that in the HMC0 group (group not fed HMC). This suggests that feeding a diet containing HMC is beneficial for rumen fermentation, which may be related to the higher rumen digestibility of HMC. During the production of HMC, the starch-protein matrix in corn kernels is disrupted, increasing the contact area between starch and rumen microorganisms. This enhances the starch digestibility of HMC in the rumen, thereby promoting microbial activity and reproduction within the rumen.

### 4.4 Effects of feeding HMC on ruminal metabolism in young Simmental bulls

#### 4.4.1 Lipid metabolism

LPC is a hydrolysis product of PC. Different types of LPC vary in carbon chain length and number of double bonds, and play a key role in lipid metabolism in living organisms (39, 40). In this study, comparative metabolomic analysis of rumen fluid between the HMC0 and HMC45 groups revealed upregulated LPC levels alongside downregulated PC. These differential metabolites were primarily enriched in five metabolic pathways: glycerophospholipid metabolism, linoleic acid metabolism, alpha-linolenic acid metabolism, choline metabolism in cancer, and retrograde endocannabinoid signaling. This suggests their involvement in rumen microbial lipid metabolism, where they may maintain a dynamic equilibrium to influence microbial growth and metabolic activity. Although studies by Wang et al. (41) and Heimerl et al. (42) reported that LPC may be associated with obesity and energy metabolism, with LPC levels negatively correlated with body mass index and energy metabolism indicators, the present study observed positive production effects (e.g., increased average daily gain) accompanying elevated LPC in the unique rumen fermentation system. This indicates that LPC may function as an important signaling molecule or an intermediate in microbial membrane remodeling, participating in the regulation of microbial community succession and metabolic activity under rapid fermentation conditions. For instance, an elevated LPC/PC ratio may reflect accelerated turnover of microbial membrane lipids to adapt to the altered fermentation environment. Concurrently, these phospholipid molecules might indirectly modulate nutrient absorption and transport by influencing the membrane structure and function of rumen epithelial cells (43). Therefore, feeding 45% HMC resulted in changes in the rumen microbial community structure, which were more conducive to the absorption of nutrients in the diet.

However, the specific role of LPC in rumen metabolism remains unclear. Studies by Wang et al. (41)and Heimerl et al. (42) found that LPC may be associated with obesity and energy metabolism, with LPC levels showing a negative correlation with increases in body mass index and energy metabolism values. In our study, through omics comparative analysis of rumen fluid differential metabolites between the control group (HMCO group, i.e., the group without added high-moisture corn) and the 45% HMC-added group (HMC45 group), several phospholipid molecules in the HMC45 group were found to exhibit partial upregulation and partial downregulation. These phospholipid molecules were primarily enriched in glycerophospholipid metabolism, linoleic acid metabolism, alpha-linolenic acid metabolism, choline metabolism in cancer, and retrograde endocannabinoid signaling. It can therefore be inferred that they participate in the lipid metabolism process of rumen microorganisms and are in a state of dynamic equilibrium regulation within the rumen, thereby affecting the growth and activity of microorganisms. In addition, phospholipid molecules may also influence the absorption and transport of nutrients by regulating the membrane structure and function of rumen epithelial cells (43). Therefore, feeding 45% HMC resulted in changes in the rumen microbial community structure, which were more conducive to the absorption of nutrients in the diet.

TCDCA is the main bioactive component of animal bile acids and has multiple functions, including inducing cell apoptosis, anti-inflammation, and immune regulation (44). TCA is one of the main active ingredients in bovine and ovine bile. It regulates the body’s immune response by inducing or inhibiting apoptosis in thymocytes at different stages (45). In addition, TCA promotes fat emulsification and accelerates fat hydrolysis, which facilitates the digestion and absorption of cholesterol and fat-soluble vitamins (46). Qiu et al. (47) found that TCA can inhibit inflammatory responses, specifically by reducing capillary permeability in inflammatory tissues, suppressing inflammatory swelling, and blocking the production of inflammatory mediators (such as nitric oxide, prostaglandin E2, and histamine). Glaser et al. (48) showed that TCA can promote the breakdown and absorption of dietary fat by activating bile salt-dependent lipase and bile acid receptors, while also stimulating liver angiogenesis to prevent bile stasis. In our study, TCDCA and TCA were both upregulated in three metabolic pathways: bile secretion, primary bile acid biosynthesis, and cholesterol metabolism. It is therefore inferred that they can regulate the community structure and function of rumen microorganisms, promote the digestion and utilization of lipids in feed, and thereby influence the rumen fermentation process. Additionally, TCDCA and TCA, as bile acids, can promote bile secretion, reduce liver burden, and have a certain protective effect on the liver (49, 50). Their upregulation as differential metabolites indicates that feeding 45% HMC may enhance lipid metabolic efficiency, maintain liver health, and thereby contribute to sustaining the host’s health and improving ruminant production performance.

#### 4.4.2 Metabolism of protein and amino acid substances

In the rumen environment, amino acids are key precursors for protein and peptide synthesis and can regulate certain metabolic pathways (51). Among these, glutamic acid is the largest contributor to the intermediate substrates of the tricarboxylic acid cycle, and dietary composition may alter the catabolic metabolism of L-glutamic acid in rumen epithelial cells via the tricarboxylic acid cycle (52). In our study, L-glutamic acid was significantly upregulated in multiple differentially regulated metabolic pathways, including taste transduction, the FoxO signaling pathway, central carbon metabolism in cancer, and alanine, aspartate, and glutamate metabolism. This suggests that feeding 45% HMC promotes the synthesis and accumulation of L-glutamic acid within rumen epithelial cells. Chen et al. (53) found through *in vitro* fermentation experiments that the increase in L-glutamic acid provided synthetic substrates for rumen microorganisms, enhanced amino acid and carbon metabolism pathways, and provided an additional energy source for microbial protein synthesis. In addition, L-glutamic acid can be degraded by microorganisms to produce ammonia, which can then be utilized by rumen microorganisms to synthesize bacterial proteins (54). It can therefore be inferred that feeding 45% HMC not only promotes the efficient conversion of L-glutamic acid in multiple metabolic pathways, but may also have a positive impact on the growth, maintenance, and function of rumen epithelial cells by optimizing energy metabolism and nitrogen utilization efficiency, thereby improving the overall production performance and health status of animals.

*γ*-aminobutyric acid (GABA) is a four-carbon non-protein amino acid that can be produced through the enzymatic decarboxylation of glutamic acid (55). When used as an additive, GABA has multiple effects, including improving animal growth performance, increasing feed intake, reducing disease incidence, and alleviating heat stress in ruminants (56, 57). Wang et al. (55) investigated the effect of GABA on rumen fermentation characteristics in ruminants through *in vitro* rumen fermentation experiments. Their study found that the addition of GABA significantly increased the dry matter degradation rate of high-fiber feed while altering the gas production pattern. This suggests that GABA may improve fiber degradation efficiency by influencing the rumen microbial community or its metabolic pathways. In our study, GABA was significantly upregulated in two differentially regulated metabolic pathways: taste transduction and alanine, aspartate, and glutamate metabolism. This suggests that feeding 45% HMC can enhance the rumen microorganisms’ ability to degrade fiber, promote microbial balance in the rumen, and thereby help maintain a stable rumen environment.

#### 4.4.3 Metabolism of other substance

Saccharin acid is an important intermediate product in the rumen fermentation process. It can generate propionic acid through decarboxylation, thereby providing energy for ruminants (58, 59). At the same time, succinic acid, as an intermediate product of the tricarboxylic acid cycle, participates in cellular energy metabolism (60). Our research found that the differential metabolite saccharinic acid was significantly upregulated in the metabolic pathways of taste transduction, central carbon metabolism in cancer, and alanine, aspartate, and glutamate metabolism. This suggests that experimental cattle fed a diet containing 45% HMC can effectively promote the production of succinic acid in the rumen. These findings indicate that feeding 45% HMC effectively enhanced succinate production in the rumen, thereby increasing its metabolic potential as a precursor for propionate. However, the analysis of ruminal contents showed no significant increase in PA concentration. This suggests that the produced PA was likely either more efficiently absorbed into the portal circulation for systemic energy use or rapidly utilized in other microbial metabolic processes. Consequently, the elevated levels of succinate metabolites may optimize ruminal fermentation efficiency. This mechanism favors enhancing the host’s energy supply and improving overall energy utilization efficiency in ruminants, rather than merely increasing the static concentration of PA within the rumen.

ADP is a high-energy phosphoric acid compound found in living organisms, typically formed by the hydrolysis of adenosine triphosphate (ATP) and the loss of a phosphate group. AMP is the final product of the hydrolysis of both ATP and ADP. ADP and AMP undergo continuous dynamic conversion within biological organisms, jointly maintaining the equilibrium of energy metabolism (61). ATP is an essential energy substance for cells to maintain normal function, whether in the synthesis of fatty acids or in glycolysis. At the same time, ADP and AMP, as intermediate products of energy metabolism, participate in these processes (62). In this study, ADP and AMP were identified as metabolically distinct substances in the rumen of the HMC45 group fed 45% HMC, exhibiting significantly upregulated expression in taste transduction and the FoxO signaling pathway. This may indicate that energy conversion in the rumen becomes more frequent after feeding 45% HMC. This is because ADP and AMP can serve as raw materials for ATP synthesis, re-synthesizing ATP under energy-sufficient conditions to ensure cellular energy supply (62). Therefore, increasing ADP and AMP helps regulate energy metabolism in the rumen and enables the rumen to obtain energy and nutrients from feed more efficiently.

This study utilized metabolomics technology to analyze the nutrient enrichment in the rumen of Simmental bulls. We found that the differential metabolites in the rumen fluid of bulls fed HMC were predominantly enriched in metabolic pathways related to lipid metabolism, leading us to speculate that HMC feeding may promote fat deposition in the bulls. Currently, HMC is relatively widely used in large-scale, intensive dairy farms (e.g., in Heilongjiang, Inner Mongolia, and Hebei); however, in beef cattle farming, with the exception of some ultra-large-scale fattening operations, its application remains limited, and traditional dry corn crushing or rolling remains the mainstream practice. Nevertheless, this study did not collect rumen fluid samples at multiple time points for dynamic monitoring of fermentation parameters, which limits our ability to more accurately elucidate the kinetic characteristics and sustained effects of different HMC substitution levels on rumen fermentation in the animals. Additionally, the study did not include an evaluation of beef quality, preventing a comprehensive assessment of the overall impact of HMC substitution levels on final farming economic benefits and meat quality. In light of these limitations, our team is currently conducting follow-up fattening trials. These trials will involve dynamic sampling of rumen fluid and systematic carcass and meat quality assessments, with the aim of comprehensively investigating the effects of HMC on rumen fermentation, fat deposition, and meat quality in Simmental bulls. The goal is to provide a more solid theoretical foundation for the scientific application of this technology in beef cattle production.

## 5 Conclusion

Our research indicates that feeding HMC at a replacement level of 45% significantly improves final weight gain, ADG, ADFI, G/F, and economic benefits in young Simmental bulls. At the same time, this feeding method can lower rumen pH and increase the relative abundance of dominant bacterial groups in the rumen at the phylum and genus levels (such as Bacteroidota, Firmicutes, *Prevotella*, *Fibrobacter*, and *Ruminococcus*). In addition, it can effectively promote the metabolism of nutrients such as lipids, proteins, and amino acids, thereby promoting the growth and development of bulls. In summary, our study investigated the appropriate replacement level of HMC in bull rearing, providing a fundamental basis for its application in subsequent production practices and fattening trials.

## Data Availability

The original contributions presented in the study are publicly available. This data can be found here: NCBI, accession number PRJNA1420153.

## Footnotes

1
www.r-project.org

2
http://www.kegg.jp/kegg/compound/

3
http://www.kegg.jp/kegg/pathway.html
